# Changes in biological productivity associated with Ningaloo Niño/Niña events in the southern subtropical Indian Oceanin recent decades

**DOI:** 10.1038/srep27467

**Published:** 2016-06-08

**Authors:** Sandeep Narayanasetti, P Swapna, K Ashok, Jyoti Jadhav, R Krishnan

**Affiliations:** 1 Centre for Climate Change Research, Indian Institute of Tropical Meteorology, Pune 411008, India; 2 Centre for Earth and Space Sciences, University of Hyderabad, Hyderabad 500046, India

## Abstract

Using observations and long term simulations of an ocean-biogeochemical coupled model, we investigate the biological response in the southern subtropical Indian Ocean (SIO) associated with Ningaloo Niño and Niña events. Ningaloo events have large impact on sea surface temperature (SST) with positive SST anomalies (SSTA) seen off the west coast of Australia in southern SIO during Ningaloo Niño and negative anomalies during Niña events. Our results indicate that during the developing period of Ningaloo Niño, low chlorophyll anomaly appears near the southwest Australian coast concurrently with high SSTA and vice-versa during Niña, which alter the seasonal cycle of biological productivity. The difference in the spatiotemporal response of chlorophyll is due to the southward advection of Leeuwin current during these events. Increased frequency of Ningaloo Niño events associated with cold phase of Pacific Decadal Oscillation (PDO) resulted in anomalous decrease in productivity during Austral summer in the SIO in the recent decades.

Changes in biological productivity have impact on the world climate. The subtropical southern ocean region is low in biological productivity, owing to the lack of supply of nutrients to surface layer. The warm surface layer in subtropics creates a large density gradient which inhibits the vertical mixing of nutrient-waters from deeper ocean^1^. High concentration of phytoplankton is observed in the southern hemisphere along Africa and South America due to equator ward flowing surface currents. In contrast to the west coasts of southern hemispheric continents, the biological productivity off the west coast of Australia is modulated by the tropical, nutrient-poor southward flowing Leeuwin Current^2^ (LC). The LC originates to the north of the North West Cape in Western Australia and transports warm tropical waters to the south^3^,^4^,^5^. The changes in LC have large impact on the marine environment. The LC is largely responsible for the transport of tropical marine species down the west coast and across into the Great Australian Bight, and enables reef-building corals to exist at the Abrolhos Islands, the highest latitude for any true corals. LC is also responsible for the occurrence of tropical fauna and flora in southern Australian waters at normally temperate latitudes^6^,^7^,^8^,^9^,^10^. In years, when the flow of the LC is relatively strong, a higher proportion of larval lobsters return to coastal waters over a larger area^11^. Recognising the effect of the LC on the catches from coastal fish stocks is vital for effective fisheries management^3^,^12^,^13^.Despite the significant impact of LC on marine living species, in depth understanding of biological productivity with LC variability is lacking.

Inter-annual and decadal variability of the LC is to a large extent driven by tropical Pacific climate variability^14^. The LC originates from eastern Indian Ocean waters and also from western Pacific Ocean (through Indonesian Through flow). The south east trade winds in the Pacific Ocean drive the South Equatorial Current westwards and advect warm surface waters towards Indonesia^4^. This results in the flow of warm, low-salinity water from the western Pacific Ocean through the Indonesian Archipelago into tropical regions of the Indian Ocean. Thus, the fluctuations in the strength of south-east trade winds in the Pacific Ocean can have impact on the strength of the LC. The LC is also influenced by El Niño, with the current weakening under El Niño and strengthening under La Niña conditions^15^.

Another important driver of LC variability is the Ningaloo events, a climate phenomenon associated with positive sea surface temperature (SST) anomalies off the west coast of Australia^16^,^17^,^18^ during Niño and negative anomalies during Niña. This phenomenon is seasonally phase-locked; it develops during October through December, reaches its peak in January-February, and decays thereafter^16^. Using long term coral data, the existence of Ningaloo Niño/Ningaloo Niña events and its impact on LC variability has been demonstrated^15^. The NOAA coral δ18O anomalies along with HadISST anomaly averaged over Ningaloo region is shown for December to February (DJF; peak phase of Ningaloo Niño/Ningaloo Niña) in Fig. 1. The HadISST anomaly and δ18O anomaly show a positive correlation (r^2^ = 0.42) which is statistically significant at 99.9% confidence level (Fig. 1) in confirmation^15^. The model SST anomaly and HadISST anomaly for the model period 1948–2009 show a positive correlation (r^2^ = 0.63) which is statistically significant at 99.9% confidence level (Fig. 1). The Ningaloo Niño and Ningaloo Niña events since 1950 are shown in Table 1. Though the impacts of Ningaloo events on the precipitation and agriculture have been studied in detail^19^, its impact on the biological productivity needs more detailed understanding. We explore the potential role of Ningaloo Niño/Ningaloo Niña in modulating the biological productivity off the coast of Australia in the southern subtropical Indian Ocean (SIO). However, it has to be noted that, not all the Ningaloo Niño events co-occur with La-Niña. Similarly, not all Ningaloo Niña events co-occur with El- Niño (shown in Table 1). The next section describes the model results and the evolution of Ningaloo Niño/Niña events associated LC and biological variability. Implication of the study and conclusion are presented in subsequent section. The final section describes the data sets and ocean general circulation model (OGCM) used for the study and methodology.

## Results

### Representation of Ningaloo events in the OGCM

Before proceeding to examining the responses of chlorophyll (Chl) to Ningaloo Niño/Niña events, we compare the model performance with the observations in terms of ocean mean state. The model reasonably captures the mean SST and Chl spatial pattern similar to the observations (Fig. S1).

To understand the evolution of Ningaloo Niño/Ningaloo Niña, we carry out composite analysis of December-February (DJF) SST anomaly (SSTA) for the Ningaloo Niño and Niña events from observation and model simulation as shown in Fig. S2. The spatial pattern of Ningaloo Niño/Niña events are well represented in the model. Though the magnitude of the simulated SST and Chl anomalies is larger than the observations, the anomaly distributions and localizations are realistic. This indicates that the model confirms the observations qualitatively.

### Biological variability associated with Ningaloo Niño (Ningaloo Niña) events

**Figure 2:**
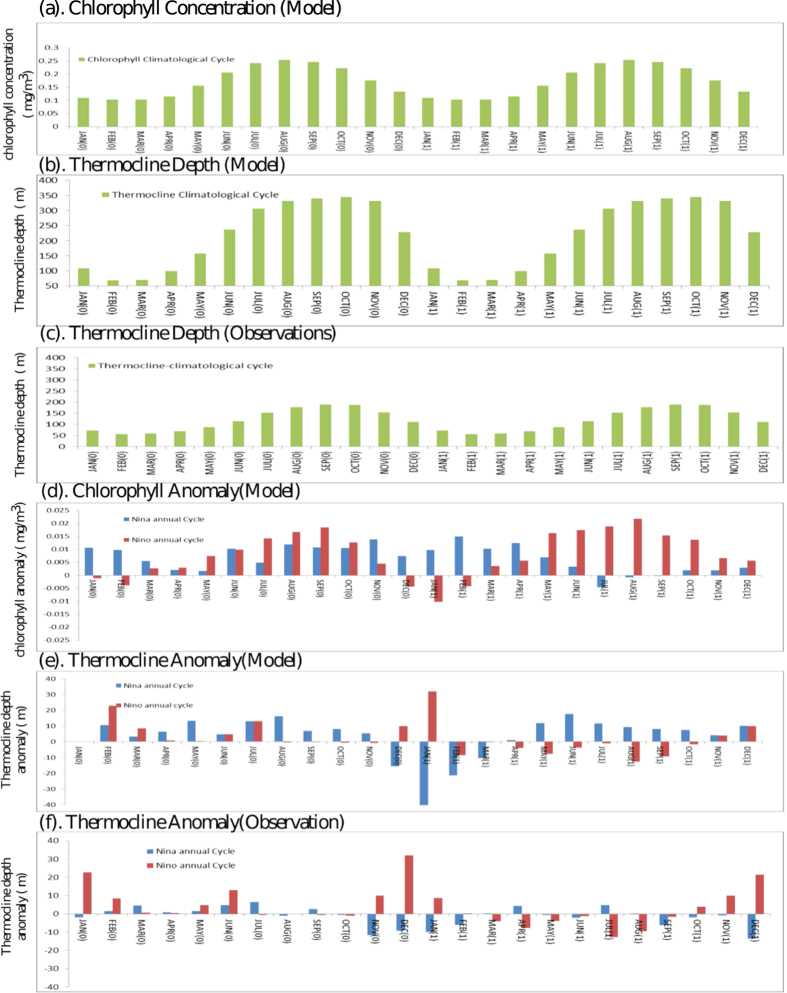
(**a**). Annual climatological cycle of chlorophyll concentration (mg/m^3^) from the model, (**b**). Annual climatological cycle of thermocline depth (m) from the model, (**c**). Same as (**b**), but from the reanalysis. (**d**). Annual cycle of chlorophyll anomalies (mg/m^3^) during Ningaloo Niño years (Red) and Ningaloo Niña years (Blue), (**e**). Annual cycle of thermocline depth anomalies (m) during Ningaloo Niño years (Red) and Ningaloo Niña years (Blue), (**f**). Same as (**e**), but from the reanalysis.

Prior to analysing the links between the Ningaloo events with the Chl variability, it is relevant to note that the peak chlorophyll along the coast of Australia occurs during June to August (JJA) as shown in the annual cycle of Chl in Fig. 2. During October to March, the LC is weaker as it flows against the strengthened low level southerly winds thus relatively weakening the downwelling, whereas during April to August the Current is stronger as the southerly winds are weaker^20^. This is reflected in the mean sea level at Fremantle (FSL). FSL serves as a proxy for the strength of LC^21^. The sea level is higher between April and August when the Leeuwin Current is stronger (lower wind stress) and lower between October and January when the current is weaker (high wind stress). Thus, although the LC flows all year round, it exhibits a strong seasonality with the stronger flows occurring during the Austral winter months (May–July). Interestingly, as the poleward-flowing LC is weaker during DJF as compared to JJA, the productivity is expected to be higher during DJF based on Ekman dynamics. However higher productivity is observed only during austral winter because of the stronger LC eddy field^22^. We have shown the climatological Chl and currents during both Austral Summer (Fig S3a) and winter (Fig S3b). Wind-driven upwelling is found to be important off west Australia coast (22^0^S)^23^.

**Figure 3:**
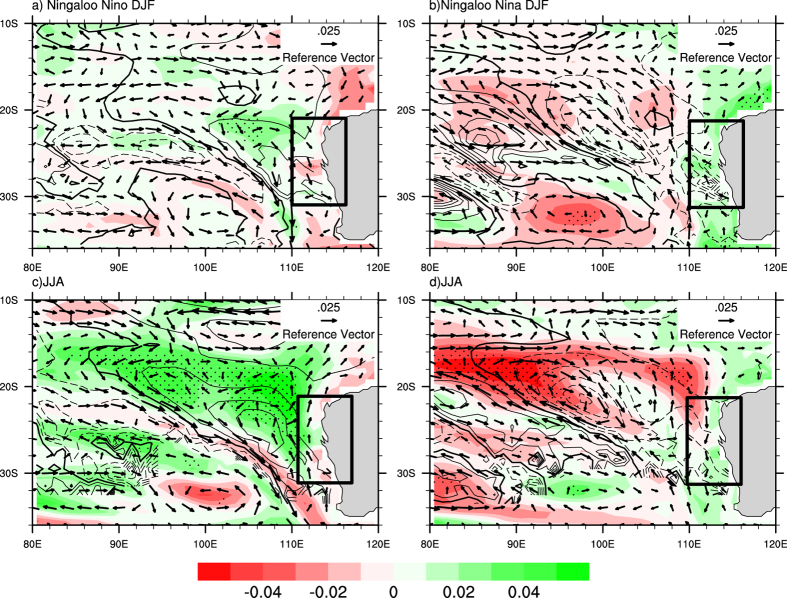
Composite plot showing chlorophyll anomalies (mg/m^3^, shaded), thermocline (m, contour) and currents (m/s, vectors) for both Ningaloo events during two different seasons DJF and JJA from the model (**a**) Ningaloo Niño (DJF) and (**b**) Ningaloo Niña (DJF); (**c**) Ningaloo Niño (JJA) and (**d**) Ningaloo Niña (JJA). Stipplings denote 95% confidence regions. This Figure is created using NCAR Command Language (Version 6.1.2) [Software]. (2013). Boulder, Colarado: UCAR/NCAR/CISL/TDD. http://dx.doi.org/10.5065/D6WD3XH5.

During a Ningaloo Niño, low Sea level Pressure Anomaly (SLPA) in the overlying atmosphere associated with positive SSTA is generated at south west coast of Australia, which anomalously increases the SLP gradient between 15°S and 32°S. The anomalous low SLP and the anomalous northerlies off the coast of west Australia amplifies the strength of the LC; the flow being poleward (Fig. 3a, Fig. S4a) causes an anomalous downwelling, which reduces the productivity at the coast, as shown in Fig. 3a. The increase in the strength of LC has different implications on each marine life species. In case of rock lobsters, higher proportion of larval lobsters return to coastal waters over a larger area when the LC is stronger^11^,^22^,^24^. While in the case of fisheries, data shows a significant negative relationship between the abundance of fishes with the strength of the LC.

During a Ningaloo Niña event, high Sea level Pressure Anomaly (SLPA) in the overlying atmosphere associated with negative SSTA is generated at south west coast of Australia, which decreases the SLPA difference between 15°S and 32°S and therefore the strength of the LC decreases. Therefore during Austral summer (DJF), when the southerly wind dominates and due the decrease in the strength of LC, anomalous upwelling is seen which results in positive chlorophyll anomaly (Chla) (Fig. 3b, Fig. S4b). The negative Chla, a proxy for the reduction in productivity during Ningaloo Niño and positive Chla, a proxy for the enhancement in productivity during Ningaloo Niña can be seen both from observation (Fig. S5) and model simulation for both the season’s summer and winter (Fig. 3). The stippling represents 95% confidence level from a two tailed student’s t-test. The lesser significance in observation owes to the lack of *in-situ* observations during Ningaloo Niño/Ningaloo Niña events.

**Figure 4:**
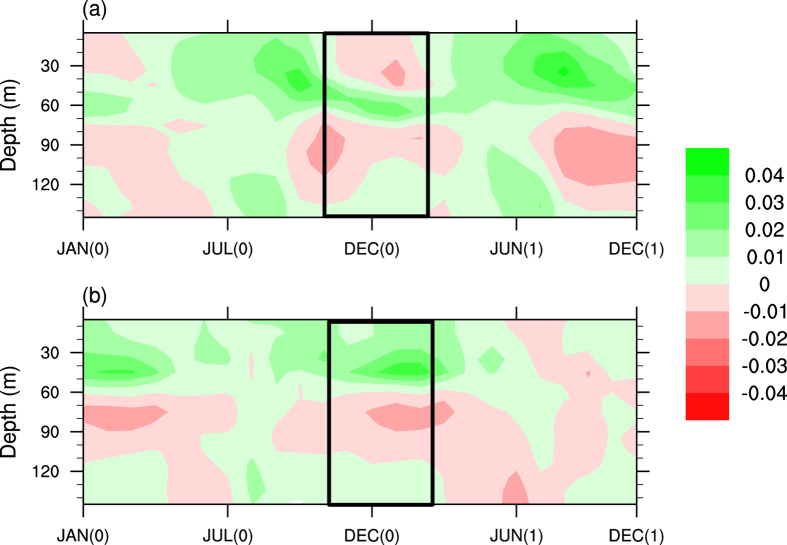
Time-depth profile of the evolution of chlorophyll anomaly (mg/m^3^) for (**a**) Ningaloo Niño and (**b**) Ningaloo Niña events from the model. This Figure is created using NCAR Command Language (Version 6.1.2) [Software]. (2013). Boulder, Colarado: UCAR/NCAR/CISL/TDD. http://dx.doi.org/10.5065/D6WD3XH5.

We further analysed the model results to find a potential mechanism behind the impacts. We have analysed the vertical profile of Chla to find the time evolution of Ningaloo Niño, Ningaloo Niña events. Figure 4a,b depicts the time evolution with depth of Chla during Ningaloo Niño/Niña composited events. The sign of chlorophyll anomalies associated with Ningaloo Niño, shows a sharp positive to negative anomalies during its peak period (December (0)-January (1)-February (1)) and then large positive anomalies during JJA (1), owing to the strength of the LC. The LC being stronger during Ningaloo Niño restricts the upwelling of nutrients and thus results in anomalous downwelling. Thus the productivity is less at the surface and the chlorophyll concentration is restricted to a depth of about 60 m. Similarly, in the case of Ningaloo Niña (Fig. 4b), the LC gets weakened and also due to the prevailing southerly winds, favours upwelling and thus the Chla is positive during the DJF in contrast to Ningaloo Niño (Fig. 4a). Thus the occurrence of Ningaloo Niño/Niña events alter the seasonal cycle of Chl variability off Australian coast, which is clearly evident from the annual cycle of Ningaloo Niño (Red) and Ningaloo Niña (Blue) events shown in Fig. 2.

### Productivity in the Leeuwin Current in Austral Summer (DJF) and Winter (JJA)

The productivity variations in the southwest coast of Australia are driven by different mechanisms during winter and summer seasons. The climatological mean LC is stronger during austral winter (JJA) as compared to austral summer (DJF). However, the interannual variations in the intensity of LC are prominent during the austral summer season and are closely linked to Ningaloo events. The intensity of the summer LC is generally found to increase during Ningaloo Niño events and weaken during Ningaloo Niña events as shown in Fig. 3 and Fig. S5. Furthermore, it is seen that enhanced summertime poleward advection by the LC associated with strengthening of the LC, tends to decrease chlorophyll concentrations (Fig. 3a) and reduced poleward advection due to weakening of LC tends to increase the chlorophyll concentration (Fig. 3b) in the southwest coast of Australia.

On the other hand, climatologically high productivity is seen during JJA (Fig S3b). The productivity variations during the austral winter are primarily linked to the variation in the eddy field strength. It can be seen that during Ningaloo Niño, the JJA season shows a higher productivity (Fig. 3c, Fig. S5c). The intensified LC during DJF restricts the Chl to nearly 60 m below surface (Fig. 4a). Similarly, during JJA after Ningaloo Niña event shows lesser productivity (Fig. 3d, Fig. S5d). The reduced intensity of LC and the prevailing southerlies bring the Chl to surface in DJF (Fig. 4b).

### Chlorophyll trends in the southern subtropical Indian Ocean and its possible association with Ningaloo Niño (Ningaloo Niña)

**Figure 5:**
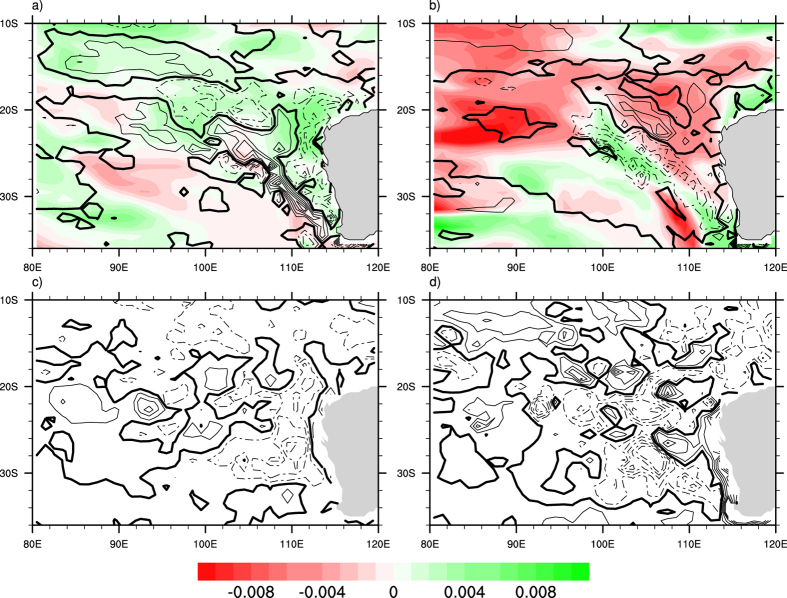
Chlorophyll trend (mg/m^3^ year^−1^, shaded) and thermocline trend (m.year^−1^, contour) for Austral Summer (DJF) during (**a**) Warm phase of Pacific Decadal Oscillation (PDO) from 1982–1997. (**b**) Cold Phase of Pacific Decadal Oscillation (PDO) from 1998–2012 from the model. Thermocline trend (m.year^−1^, contour) for Austral Summer (DJF) during (**c**). Warm phase of Pacific Decadal Oscillation (PDO) from 1982–1997. (**d**) Cold phase of Pacific Decadal Oscillation (PDO) from 1998–2012 from the reanalysis. This Figure is created using NCAR Command Language (Version 6.1.2) [Software]. (2013). Boulder, Colarado: UCAR/NCAR/CISL/TDD. http://dx.doi.org/10.5065/D6WD3XH5.

We have seen that Ningaloo Niño events can cause enhanced productivityin the southern SIO during Austral winter (June-September) and Ningaloo Niña events during Austral summer (December-February). The frequency of Ningaloo Niño events are more during the recent decades associated with cold phase of Pacific Decadal Oscillation (PDO), in agreement with Feng *et al.*^14^. They reported the association between Ningaloo Niño and PDO. Recently, Doi *et al.* 2015 also showed that the global warming and the Interdecadal Pacific Oscillation warm the ocean off the west coast of Australia after the late 1990s, which started driving rainfall variability regionally there. This made the rainfall predictability near the coastal region of Western Australia on a seasonal time scale drastically enhanced in the late 1990s. They have also shown that the decadal change of air-sea dynamics around the western Australia also may be important for decadal change of the productivity in the southern SIO in the recent decade^24^,^25^. The trends in Chl and thermocline are estimated for DJF for the warm and cold phases of PDO and are shown in Fig. 5. It is evident from Fig. 5 that Chl shows a decreasing trend associated with deepening trend of thermocline during the cold PDO phase as shown in Fig. 5b. Similarly, during the warm PDO phase the Chl anomalies are increasing associated with shoaling trend in the thermocline (Fig. 5a). Similar features can be seen from the observations as shown in Fig. 5c,d. Since the Chl observations are not available for the warm PDO phase, we have only shown the thermocline trends from the reanalysis data. These suggest that biological productivity in the southern SIO is modulated by the large scale forcing associated with PDO during Ningaloo events.

## Discussion

The Ningaloo Niño/Niña plays a key role in influencing the strength of LC, thus affecting the variability of chlorophyll close to the Australian coast, influencing the local ecosystem. According to the recent studies, the number of Ningaloo Niño events has increased since late 1990s. In this context, we examine the chlorophyll response to both the Ningaloo Niño and Ningaloo Niña events. We use both observation and long-term simulation from ocean biogeochemical coupled model which is forced by interannual forcing for the period 1948 to 2009.

Evaluations of the model results show that the ocean model is able to reasonably simulate the Ningaloo Niño/Niña events in terms of spatial patterns and temporal evolution. The model also captures the seasonal variation of the LC and chlorophyll variability during Austral summer and winter as shown in Fig. 3. The results show that during Ningaloo Niño events, positive SSTA during DJF strengthens the Leeuwin Current. This results in the decrease in the chlorophyll anomaly off the coast of west Australia in southern subtropical Indian Ocean. However during Ningaloo Niña events, the negative SSTA during DJF decreases the strength of the LC, which results in enhanced upwelling and increase in the chlorophyll anomaly.

An analysis of long term observational SST and δ18O coral data and model simulations indicates that the number of Ningaloo Niño events have increased during the cold phase of PDO, which confirms the conjecture by Feng *et al.*^14^. More number of Ningaloo Niño (Niña) events during the cold (warm) phase of PDO alters the annual cycle by enhancement (reduction) of chlorophyll concentration during DJF. This explains the apparent decreasing trend in the DJF productivity during the cold phase. Similarly during warm phases of PDO, there was an increase in the trend in DJF productivity. The results indicate that increased frequency of Ningaloo Niño events associated with cold phase of PDO has resulted in anomalous decrease in productivity and increased frequency during Niña events associated with warm phase of PDO resulted in anomalous increase in productivity during Austral summer in the southern SIO in the recent decades.

### Data

We have used the Hadley Centre Global Sea Ice and Sea Surface Temperature^26^ (HadISST) with 1° X 1° resolution from 1950 to 2011.We also use the merged observed chlorophyll concentrations, available for the period 1997–2014 (http://hermes.acri.fr/). These concentrations are derived from measurements taken using SeaWiFS, MODIS, MERIS and VIIRS sensors. We also used NOAA Ningaloo Reef coral stable isotope (δ18O) data for the period 1878–1994. We have identified the observed and simulated Ningaloo events by using the Ningaloo Niño index (NNI), defined as the monthly Sea Surface Temperature (SST) anomaly averaged over the region 113°E–116°E and 28°S–22°S^18^. We catalogue an event as a Ningaloo Niño (Ningaloo Niña) when the magnitude of December to February (DJF) NNI is above (below) one standard deviation. Thus, the Ningaloo Niño (Ningaloo Niña) years identified given in Table 1 from the model, in agreement with Feng *et al.*^17^., and Kataoka *et al.*^18^. We estimated the thermocline depth as the maximum slope (dT/dz) in a temperature-depth profile^27^. The subsurface datasets were from Simple Ocean Data Analysis (SODA)^28^ for the period 1958–2008.

### Ocean Model

The OGCM used in the present study is the Modular Ocean Model (MOM4p1 with biogeochemistry module), a hydrostatic model using Boussinesq approximation, and having a rescaled geopotential vertical coordinate^29^. Key physical parameterizations (KPP) include a surface bound which computes vertical diffusivity, vertical viscosity and non-local transport as a function of the flow and surface forcing^30^. Model has 50 levels in the vertical from surface to 5300 m; the horizontal resolution is 360 × 200 with 1° × 1° longitude and latitudinally varying grid with 0.25° near equator and 0.5° poleward of 10°. Bottom topography is represented by the partial cell method^31^,^32^. The biogeochemical model for the simulation of chlorophyll concentration is the Tracers of Phytoplankton with Allometric Zooplankton (TOPAZ) model^33^,^35^. The biogeochemical model TOPAZ developed at GFDL has been coupled with MOM4p1. This prognostic ocean biogeochemistry model contains 25 tracers including three phytoplankton groups (diatoms, eukaryotic phytoplankton, diazotrophs), two forms of dissolved organic matter (labile and semi-labile), heterotrophic biomass, detritus, nutrients (N, P, Si, and Fe), dissolved inorganic carbon and alkalinity, dissolved inorganic species for coupled C, N, P, Si, Fe, CaCO_3_, O_2_, and lithogenic cycling with flexible N:P:Fe stoichiometry^34^,^35^,^36^. Further details of the model can be availed from Griffies *et al.* and Dunne *et al.*^29^,^34^. The model has been spun up, and subsequently integrated for 120 years to reach a steady state, by initializing the model with the annual climatologies of temperature and salinity from Levitus^36^,^37^ and forced with climatological forcing derived from CORE (Common Ocean-ice Reference Experiments). From this steady state, we carried out an interannual integration for a 62-years using Corrected Interannual Forcing (CIAF) for the 1948–2009 period. Our analysis of model results pertains to the data from this 62-year simulation^38^.

## Additional Information

**How to cite this article**: Sandeep, N. *et al.* Changes in biological productivity associated with Ningaloo Niño/Niña events in the southern subtropical Indian Ocean in recent decades. *Sci. Rep.* **6**, 27467; doi: 10.1038/srep27467 (2016).

## Supplementary Material

Supplementary Information

## Figures and Tables

**Figure 1:**
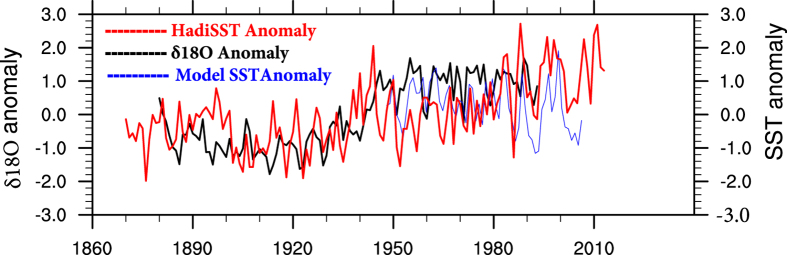
Time series showing the evolution of δ18O Anomaly (ppm, black) versus HadiSST anomaly (K, red) and Model SST anomaly (K, blue).

**Table 1:** Classification of Ningaloo Niño and Ningaloo Niña events from the model.

Ningaloo Niño	Ningaloo Niña
1955–56	1951–52
1960–61	1952–53
1961–62	1953–54
1962–63	1986–87
1966–67	1990–91
1973–74	2003–04
1976–77	2004–05
1979–80	2005–06
1982–83	
1996–97	
1999–00	
2010–11	
