# Supplementary material for: Changes in biological productivity associated with Ningaloo Niño/Niña events in the southern subtropical Indian Oceanin recent decades
Source: Sci Rep. 2016 Jun 8;6:27467. doi: 10.1038/srep27467 (PMC4897695; doi:10.1038/srep27467)
Supplement: Supplementary Information [file srep27467-s1.pdf]

Changes in biological productivity associated with Ningaloo Niño / Niña events in the southern subtropical Indian Ocean in recent decades.

Sandeep N[1*], Swapna P[1], Ashok K[1, 2], Jyoti J[1] and R Krishnan[1]

[1] Centre for Climate Change Research, Indian Institute of Tropical Meteorology, Pune 411008, India

[2] Centre for Earth and Space Sciences, University of Hyderabad, Hyderabad 500046, India

***Corresponding Author address:**

Mr. Sandeep Narayanasetti

Centre for Climate Change Research,

Indian Institute of Tropical Meteorology,

Pune 411008, INDIA.

Tel: +917875323409

Email: sandeep.cat@tropmet.res.in

Supplementary Figures:

Figure S1: Spatial map showing Climatology of Sea Surface Temperature (^{0}C) for a) Observation; b) Model; Spatial map showing Climatology of Chlorophyll (mg/m^3) for c) Observation; and d) Model. This Figure is created using NCAR Command Language (Version 6.1.2) [Software]. (2013). Boulder, Colarado: UCAR/NCAR/CISL/TDD. http://dx.doi.org/10.5065/D6WD3XH5.

Figure S2: Spatial map showing Sea Surface Temperature (SST) anomalies (^{0}C shaded) for both Ningaloo events a) Ningaloo Niño; b) Ningaloo Niña for observations; c) Ningaloo Niño and d) Ningaloo Niña for Model. Stipplings denote 95% confidence regions. This Figure is created using NCAR Command Language (Version 6.1.2) [Software].(2013). Boulder,Colarado: UCAR/NCAR/CISL/TDD. UCAR/NCAR/CISL/TDD. http://dx.doi.org/10.5065/D6WD3XH5.

Figure S3: Plot showing climatological chlorophyll concentration (mg/m^3) during a) AustralSummer (DJF) and b) Austral winter (JJA). Vectors represent the current (m/s) and contoursrepresent the depth of the thermocline (m). This Figure is created using NCAR Command Language (Version 6.1.2) [Software]. (2013). Boulder, Colarado: UCAR/NCAR/CISL/TDD. http://dx.doi.org/10.5065/D6WD3XH5.

Figure S4: Composite plot showing Wind vectors (m/s) composite for December to February (DJF) overlaid on Sea level Pressure (mb, contour) for both a) Ningaloo Niño and b) Ningaloo Niña. This Figure is created using NCAR Command Language (Version 6.1.2) [Software]. (2013). Boulder, Colarado: UCAR/NCAR/CISL/TDD. http://dx.doi.org/10.5065/D6WD3XH5.

Figure S: Composite plot showing chlorophyll anomalies (mg/m^3, shaded) and thermocline (m, contour lines) for both Ningaloo events during two different seasons DJF and JJA from the model a)

Ningaloo Niño (DJF) and b) Ningaloo Niña (DJF); c) Ningaloo Niño (JJA) and d) Ningaloo Niña (JJA) from the observations. Vectors represent the currents (m/s). Stipplings denote 95% confidence regions. This Figure is created using NCAR Command Language (Version 6.1.2) [Software]. (2013). Boulder, Colarado: UCAR/NCAR/CISL/TDD. http://dx.doi.org/10.5065/D6WD3XH5.

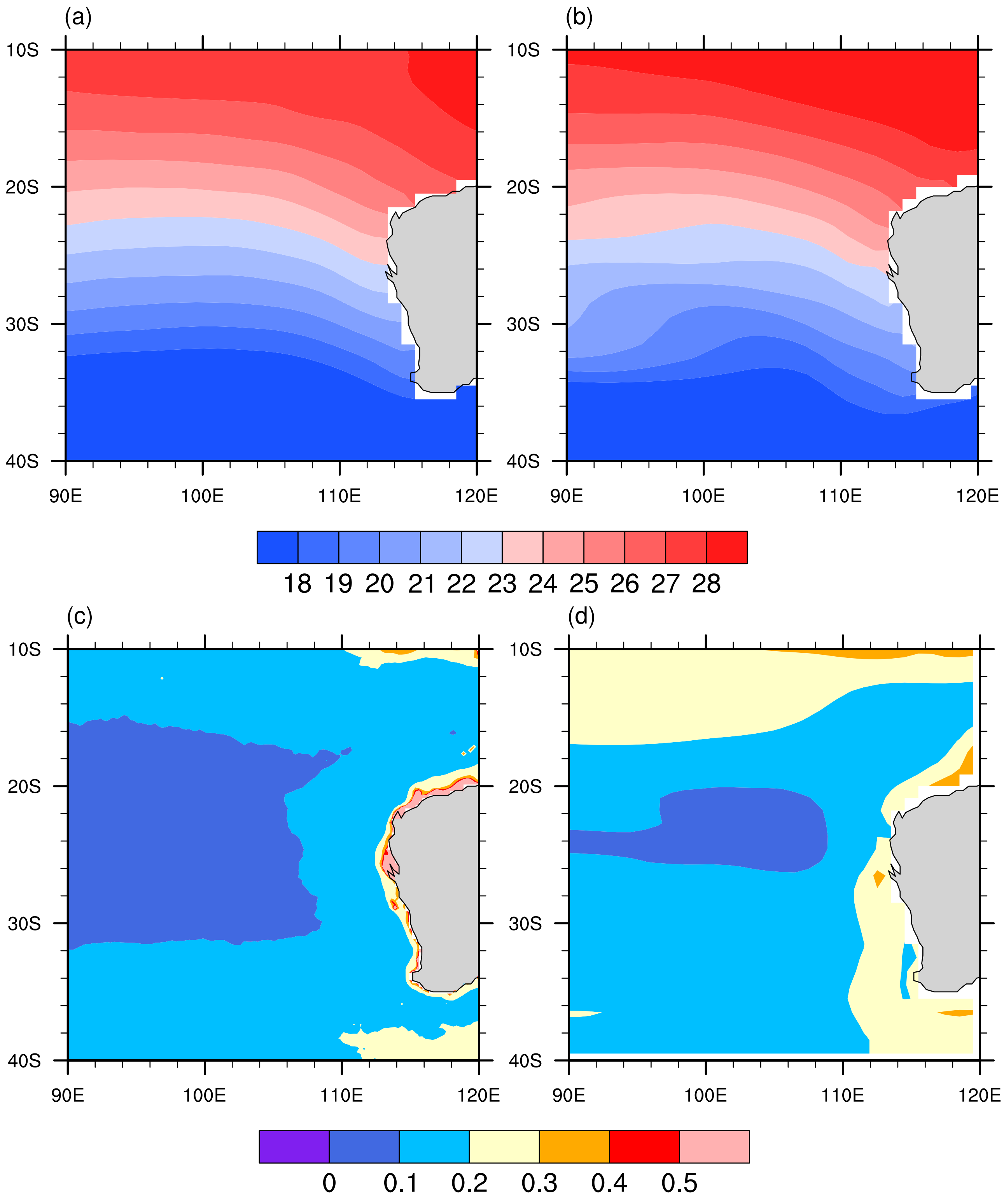
(a)
(b)
10S
20S
30S
40S
90E
100E
110E
120E
18 19 20 21 22 23 24 25 26 27 28
(c)
(d)
0 0.1 0.2 0.3 0.4 0.5

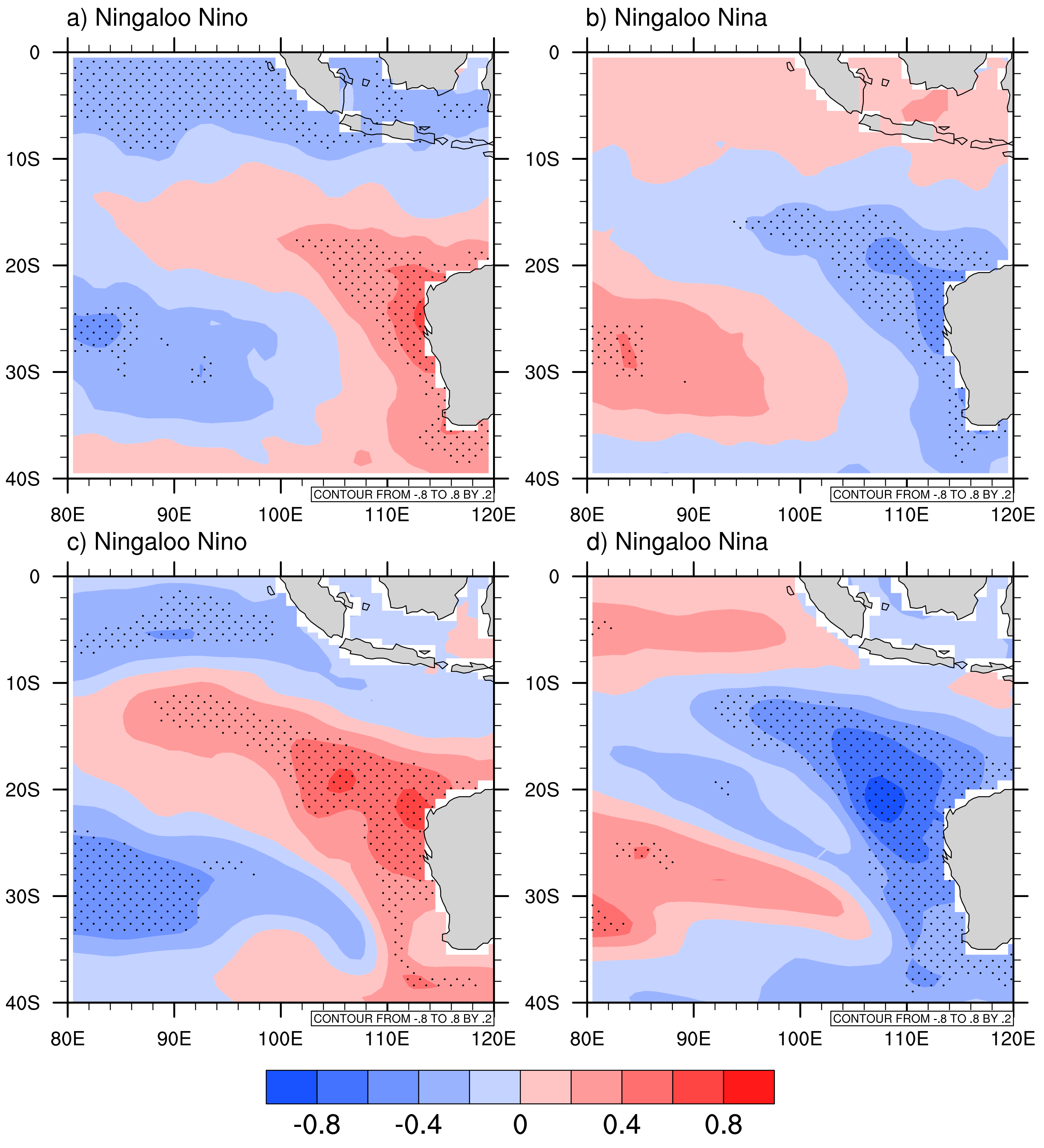

a) Ningaloo Nino
b) Ningaloo Nina
c) Ningaloo Nino
d) Ningaloo Nina
0
10S
20S
30S
40S
80E
90E
100E
110E
120E
CONTOUR FROM -.8 TO .8 BY .2
-0.8
-0.4
0
0.4
0.8

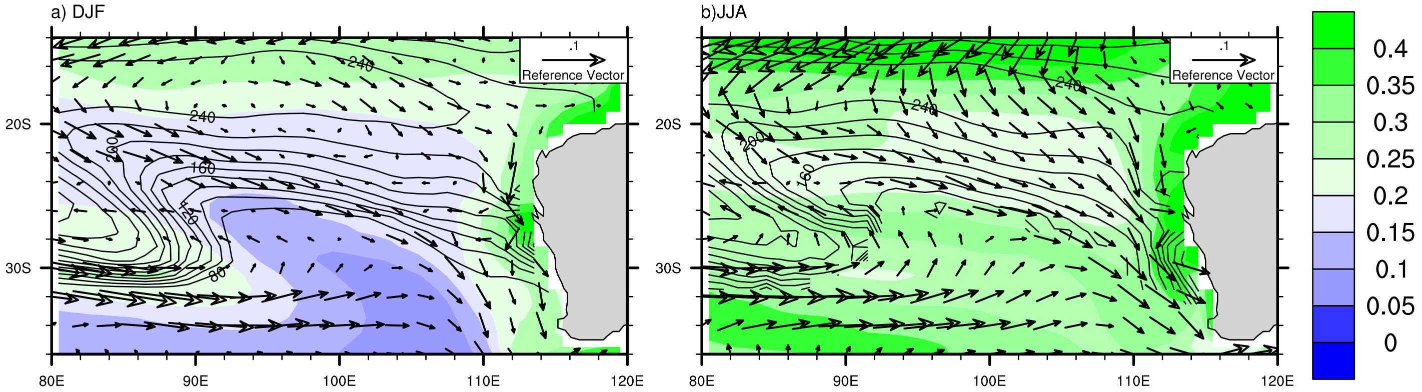

a) DJF
b)JJA
.1
Reference Vector
240
200
160
120
80
20S
30S
80E
90E
100E
110E
120E
0.4
0.35
0.3
0.25
0.2
0.15
0.1
0.05
0

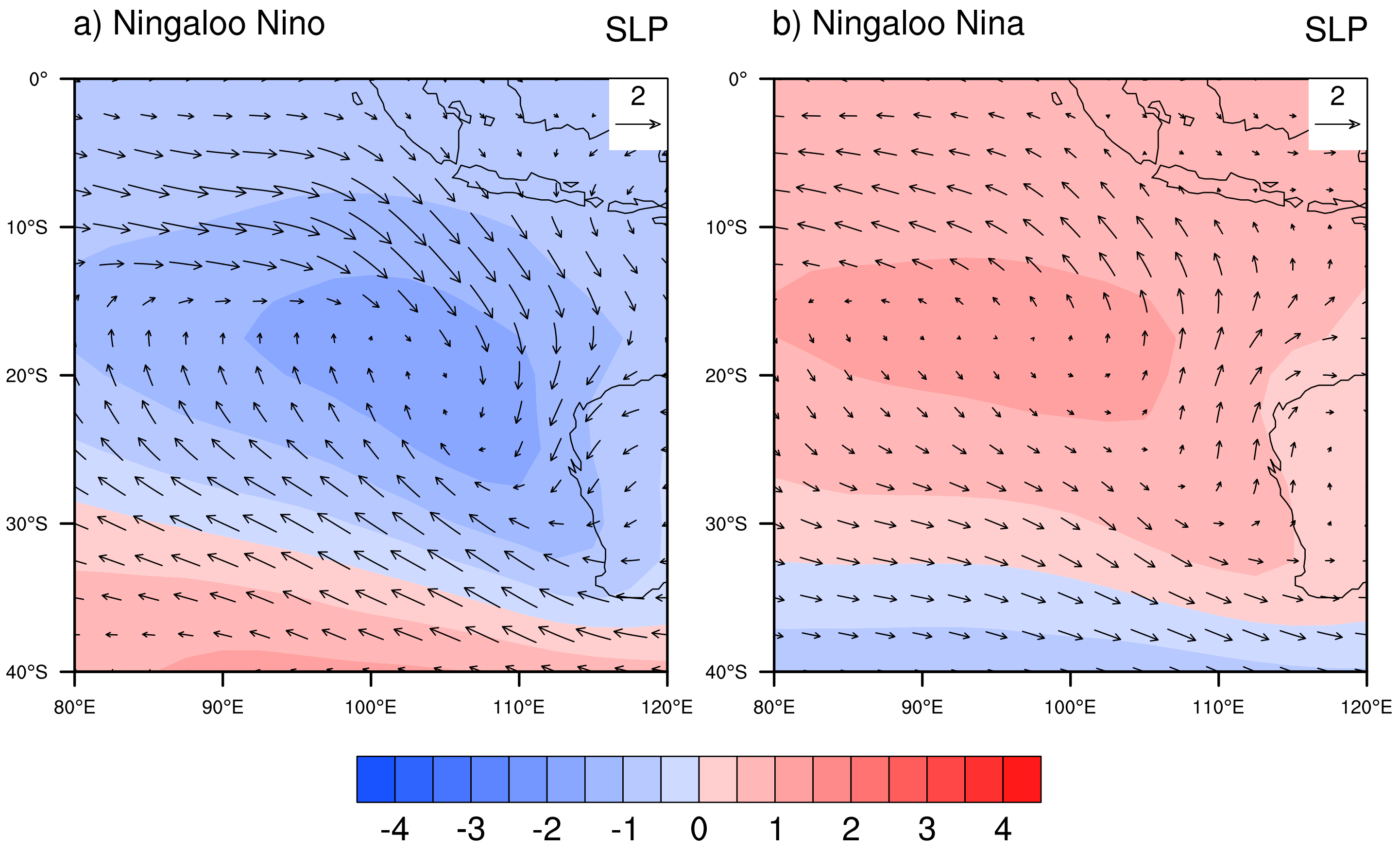

a) Ningaloo Nino
SLP
b) Ningaloo Nina
SLP
2
2
0°
10°S
20°S
30°S
40°S
80°E
90°E
100°E
110°E
120°E
-4
-3
-2
-1
0
1
2
3
4

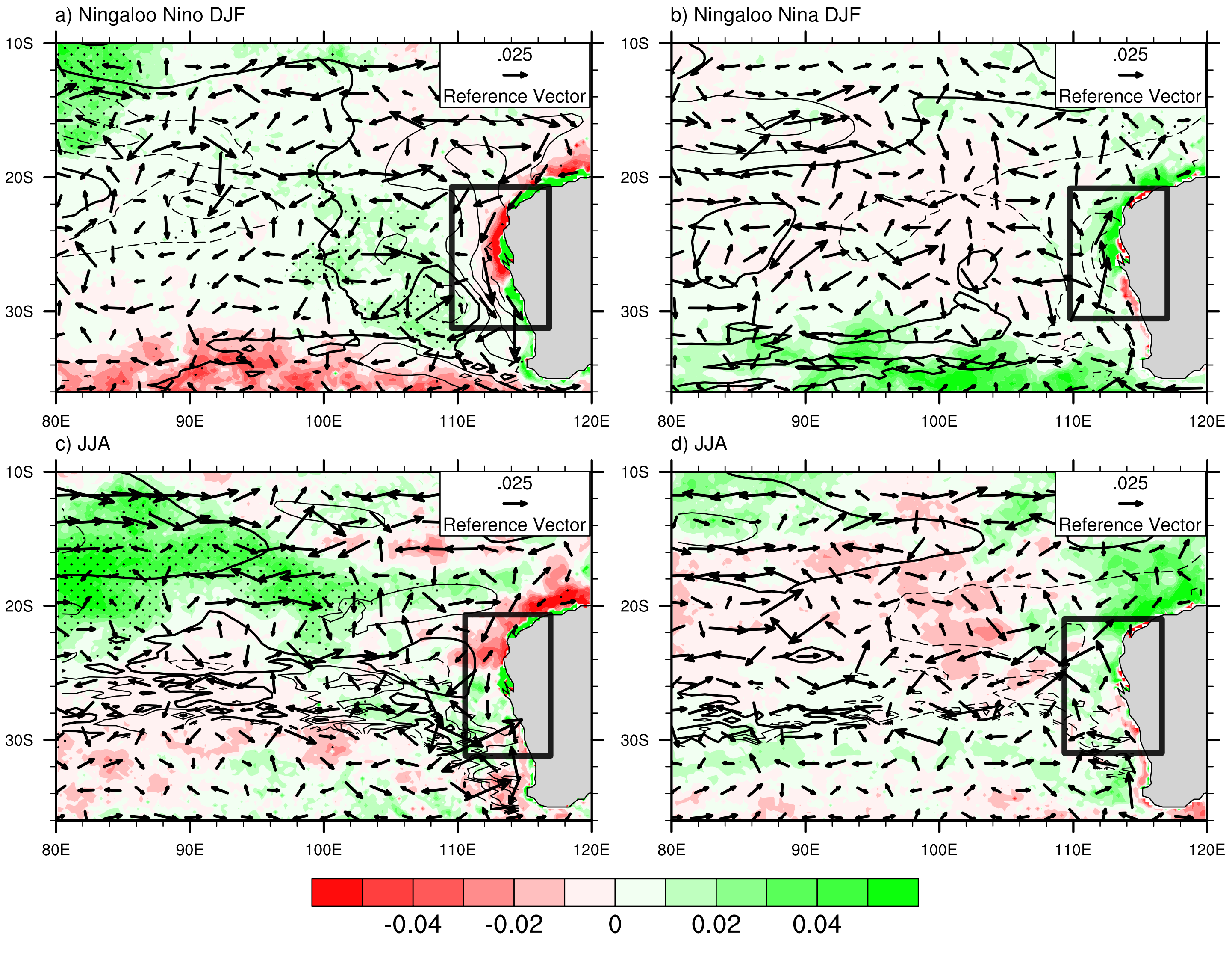

a) Ningaloo Nino DJF
b) Ningaloo Nina DJF
c) JJA
d) JJA
.025
Reference Vector
10S
20S
30S
80E
90E
100E
110E
120E
-0.04
-0.02
0
0.02
0.04
